# Kavanah Project: beyond health promotion

**DOI:** 10.31744/einstein_journal/2024CE1147

**Published:** 2024-09-20

**Authors:** Leandro Luongo Matos, Sidney Klajner

**Affiliations:** 1 Hospital Israelita Albert Einstein Faculdade Israelita de Ciências da Saúde Albert Einstein São Paulo SP Brazil Faculdade Israelita de Ciências da Saúde Albert Einstein, Hospital Israelita Albert Einstein, São Paulo, SP, Brazil.; 2 Hospital Israelita Albert Einstein São Paulo SP Brazil Hospital Israelita Albert Einstein, São Paulo, SP, Brazil.

Dear Editor,

In late 2021, two driven medical students from *Faculdade Israelita Albert Einstein* (FICSAE) in São Paulo, Brazil, initiated the "Kavanah Project."^(1)^ This initiative targets the improvement of healthcare access by conducting surgical missions in areas with long wait times for medical procedures, a prevalent issue in Brazil.^(2,3)^ Diverging from conventional approaches, *Kavanah*, meaning "direction," "intention," or "purpose" in Hebrew, underscores a deliberate method of medical assistance, echoing deeper institutional principles of good deeds (*Mitzvá*), health (*Refuá*), education (*Chinuch*), and social justice (*Tsedaká*).

The project operates on three core pillars: social outreach, health management enhancement, and educational empowerment. The social aspect focuses on bolstering patient accessibility to superior healthcare; health management endeavors to augment the capacities of local health facilities; and education provides FICSAE medical students with avenues for surgical exposure and the cultivation of empathy, teamwork, and leadership skills. These pillars seamlessly integrate with the core values of the institution, offering a comprehensive approach to medical education and service provision.

Two medical expeditions were conducted in the rural landscapes of Minas Gerais and São Paulo, two southeastern states in Brazil. These missions involved 88 dedicated volunteers, including 27 physicians (comprising 15 general surgeons or gynecologists, 11 anesthesiologists, and one primary care physician), 4 nurses, 5 nursing technicians, 2 scrub nurses, 3 engineers, and 47 medical students. During these missions, 64 patients received treatment, and 70 surgeries were performed. Though modest in scale, these efforts signify the inaugural strides of a pivotal initiative. If replicated nationwide, such endeavors could enable medical students to directly contribute to public health, under the mentorship of seasoned physicians, from the onset of their medical training.

Initially, the Kavanah Project was integrated into FICSAE's Medical School as a mandatory extension program by Resolution 7/2018 of the Brazilian Ministry of Education.^(4)^ This allows students from different courses to participate, enhancing their technical skills and socio-emotional development. The project has expanded its impact and professionalism to serve as a model for other medical schools with the support of the hospital's leadership. We are proud of our students. This project exemplifies how medical education can incorporate learning, social responsibility, and the promotion of equitable access to high-quality care.
